# IgE-Mediated Legume Allergy in Children: Insights from a Single-Center Experience in Italy

**DOI:** 10.3390/nu18111810

**Published:** 2026-06-04

**Authors:** Beatrice Serra, Simona Barni, Claudia Valleriani, Beatrice Coppadoro, Francesco Catamerò, Letizia Ciliberti, Mattia Giovannini, Giulia Liccioli, Lucrezia Sarti, Leonardo Tomei, Antonella Muraro, Francesca Mori

**Affiliations:** 1 Food Allergy Referral Centre, Veneto Region, Department of Women’s and Children’s Health, Padua University Hospital, 35128 Padua, Italy; bea.s.93@hotmail.it (B.S.); muraro@centroallergiealimentari.eu (A.M.); 2 Allergy Unit, Meyer Children’s Hospital IRCCS, 50139 Florence, Italy; simonabarni@hotmail.com (S.B.); letizia.ciliberti@unifi.it (L.C.); mattiag88@hotmail.it (M.G.); giulia.liccioli@meyer.it (G.L.); lucrezia.sarti@meyer.it (L.S.); leonardo.tomei@meyer.it (L.T.); francesca.mori@meyer.it (F.M.); 3 Immunology Laboratory, Meyer Children’s Hospital IRCCS, 50139 Florence, Italy; claudia.valleriani@meyer.it; 4 Department of Women’s and Children’s Health, University of Padua, 35128 Padua, Italy; beatrice.coppadoro@gmail.com; 5 Department of Health Sciences, University of Florence, 50139 Florence, Italy

**Keywords:** legume allergy, pediatric food allergy, pea allergy, retrospective study, oral immunotherapy, non-priority legumes, food labeling, anaphylaxis

## Abstract

**Background/Objectives**: Legume allergy is increasingly recognized as plant-based diets expand and legume proteins are widely used in processed foods. We aimed to characterize the clinical features, sensitization profiles, and management outcomes of IgE-mediated legume allergy in Italian children. **Methods**: This retrospective single-center study (January 2022–January 2024) included children (<18 years) allergic to ≥1 index legume (pea, lentil, chickpea, common bean, or soy). Diagnosis required a compatible clinical history and evidence of IgE sensitization. Clinical and allergy characteristics were analyzed. **Results**: Fifty-five children (63.6% male) were included; all had atopic comorbidities, and 96.4% had additional food allergies. Median age at first reaction was 18 months; anaphylaxis occurred at onset in 12.7%, most frequently triggered by pea. Pea (70.9%) and lentil (69.1%) were the most prevalent allergies, with pea causing 50% of index-legume anaphylaxis. Multi-legume allergy predominated (74.5%), with frequent co-allergy among pea, lentil, and chickpea (56–86%). Soy allergy was less frequent and mainly associated with Gly m 4 sensitization. Single-legume allergy (25.5%) was associated with later onset (54 vs. 15 months; *p* = 0.013) and liver transplantation (21% vs. 2%; *p* = 0.047). Peanut co-allergy occurred in 25.5%. Among 34 oral food challenges (OFCs), 23.5% were positive, including one case of pea-induced anaphylaxis. Of 16 oral immunotherapy (OIT) protocols initiated, 31.3% reached the full target maintenance dose, 37.5% remained on a lower, partial maintenance dose, and 31.3% were discontinued due to oral allergy syndrome (OAS). **Conclusions**: Pediatric legume allergy is characterized by early onset, frequent multi-legume involvement, and common co-allergies. In this cohort, pea allergy was associated with the highest proportion of severe reactions. Species-specific differences in severity, patterns of multi-legume involvement, and OIT outcomes should be interpreted cautiously given the limited sample size, while highlighting the need for tailored management and improved risk assessment across legume species.

## 1. Introduction

Legumes are an affordable and globally important source of plant protein. In recent years, their consumption has increased markedly, driven by the growing popularity of vegetarian and vegan diets, sustainability concerns, and the expanding use of legume-derived ingredients in gluten-free and processed foods [1,2,3]. This dietary shift has occurred alongside the rising global burden of food allergy (FA) and food-induced anaphylaxis [4,5], thereby increasing the clinical relevance of IgE-mediated legume allergy, which ranges from mild localized symptoms to life-threatening anaphylaxis [6].

Despite their clinical relevance, international labeling regulations for legumes are inconsistent. Peanuts and soybeans are widely recognized as major allergens (e.g., the “Big 9” in the United States) [7], and European legislation (EU Regulation No. 1169/2011) also mandates labeling for lupine [8]. However, several “non-priority” legumes, particularly peas and lentils, have increasingly been reported as triggers of food-induced anaphylaxis [9,10] and may act as hidden allergens in processed foods, increasing the risk of accidental exposure [11,12,13,14].

The classification of “priority” legumes remains debated, as several “non-priority” legumes have been associated with persistent allergy and relatively low tolerance rates in some cohorts (20–30%) [10], suggesting a disease burden comparable to peanut and indicating that current prioritization frameworks may not fully reflect their clinical impact.

The epidemiology of legume allergy varies according to regional dietary patterns. Peanut allergy predominates in many Western countries and soy allergy is more common in East Asia, whereas lentils, chickpeas, and peas are frequent triggers in Mediterranean and South Asian populations [5,15,16,17,18]. In Mediterranean pediatric cohorts, lentils are often the leading cause of legume allergy, followed by chickpeas and peas, consistent with the frequent childhood exposure to these legumes, together with common beans, as staple dietary components in Mediterranean countries [16,17,18]. Soy also has particular relevance in pediatric nutrition because of its widespread use in infant feeding and processed foods [19,20,21]. In contrast, legumes such as lupine and faba bean have more limited and regionally variable consumption patterns [16], whereas peanut is often considered separately because of its distinct epidemiological profile and traditional dietary classification alongside tree nuts in Italy [22]. Accordingly, pea, lentil, chickpea, common bean, and soy were selected as the primary (“index”) legumes for cohort definition and analysis in the present study.

The pathophysiology of legume allergy is primarily driven by seed storage proteins, including vicilins (7S globulins), legumins (11S globulins), and 2S albumins, which are thermostable and resistant to digestion [15,23]. In this nomenclature, “S” denotes Svedberg units, reflecting the sedimentation properties of these protein fractions. The structural homology of these proteins contributes to high rates of co-sensitization and potential cross-reactivity among legumes; however, sensitization does not always translate into clinically relevant allergy [16,24,25,26].

Diagnosis is based on a compatible clinical history supported by evidence of IgE sensitization, whereas oral food challenge (OFC), a supervised diagnostic procedure involving controlled exposure to the suspected food allergen, remains the gold standard for confirmation or tolerance assessment [6,15,27,28]. Management primarily relies on avoidance and emergency preparedness, although oral immunotherapy (OIT) is emerging as a strategy to increase reaction thresholds. Nevertheless, standardized OIT protocols and real-world data remain limited for legumes other than peanut [29,30,31,32].

Emerging phenotypes such as transplant-acquired food allergy (TAFA) following liver transplantation suggest additional immune-mediated mechanisms that may contribute to the development of new food allergies in children, including legumes [33,34,35].

This study aimed to characterize a cohort of Italian children with IgE-mediated legume allergy, focusing on their clinical and allergy characteristics.

## 2. Materials and Methods

### 2.1. Study Design and Study Population

This retrospective, single-center study was conducted at the Allergy Unit of Meyer Children’s Hospital IRCCS, Florence, Italy. Medical records of patients evaluated between 1 January 2022, and 31 January 2024, were reviewed to identify individuals with IgE sensitization (serum-specific IgE [sIgE] > 0.10 kU_A_/L) to index legumes. For the purposes of this study, “index legumes” were defined as pea, lentil, chickpea, common bean, and soy, which were selected for cohort screening, eligibility assessment, and primary analyses based on their relevance in Mediterranean dietary patterns and pediatric nutrition. Peanut, faba bean, and lupine were considered non-index legumes and were therefore recorded and analyzed separately as clinically relevant co-allergies.

Eligible participants were <18 years of age at the time of evaluation and had a diagnosis of IgE-mediated allergy to at least one index legume. Diagnosis required both: (1) a clinical history consistent with an immediate allergic reaction within 2 h of exposure to the implicated index legume; and (2) evidence of IgE sensitization to the same legume, documented by positive skin testing (skin prick test [SPT] or prick-by-prick [PbP], wheal ≥ 3 mm) and/or legume-specific IgE (sIgE > 0.10 kU_A_/L).

**Figure 1:**
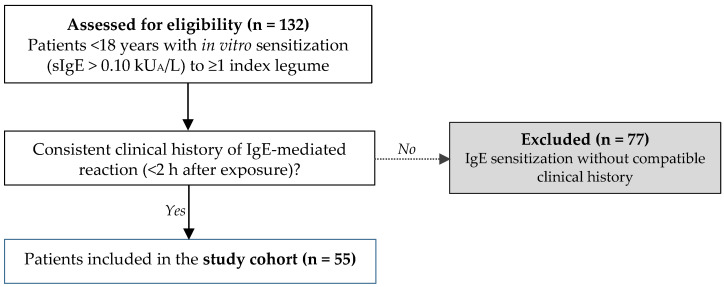
Flowchart of study population selection.

Overall, 132 children with legume sensitization (sIgE > 0.10 kU_A_/L) to at least one index legume were screened. Fifty-five patients met the inclusion criteria and were enrolled in the study cohort. The remaining 77 patients had IgE sensitization without a compatible clinical history and were excluded to minimize outcome misclassification (Figure 1).

### 2.2. Data Collection

Demographic and clinical data were extracted from electronic medical charts and supplemented, when necessary, by telephone interviews. The collected variables included:

- Demographics: Sex (male/female), date of birth, and age at study inclusion.
- Atopic history and comorbidities: Family and personal history of atopy, including food allergies, asthma, allergic rhinitis, atopic dermatitis (AD), and eosinophilic esophagitis (EoE), diagnosed according to international guidelines.
- Legume allergy history: Characteristics of the first and subsequent reactions, implicated triggers, treatments administered, and outcomes of OFCs and OIT.
- Other comorbidities: Celiac disease, drug hypersensitivity, TAFA, gastroesophageal reflux disease, autism spectrum disorder, and chronic spontaneous urticaria (CSU).
- Chronic systemic therapies: Ongoing treatment with biologic or immunosuppressive agents for comorbid conditions.
- Current management: Dietary restrictions and ongoing OIT, when applicable.
- Allergy testing data retrieved from the hospital database included legume skin testing (SPT and PbP), legume-specific IgE, total serum IgE, and aeroallergen sensitization assessed by SPT and/or specific IgE.

### 2.3. Diagnostic Procedures

#### 2.3.1. Skin Testing

SPTs were performed on the volar surface of the forearm using standardized extracts (Lofarma, Milan, Italy) for both food allergens and common aeroallergens (house dust mites [HDMs], animal dander, molds, and pollens). PbP tests were conducted using the food form most relevant to the patient’s clinical history, including cooked or canned legumes, soy milk, and fresh foods when appropriate. SPTs and PbP tests were considered positive if the wheal diameter was ≥3 mm at the 15-min reading [36,37]. Histamine (10 mg/mL; Lofarma, Milan, Italy) was used as the positive control and normal saline as the negative control.

#### 2.3.2. Serum-Specific IgE and Component-Resolved Diagnosis (CRD)

sIgE quantification was performed using the ImmunoCAP FEIA System (Thermo Fisher Scientific/Phadia, Uppsala, Sweden). CRD was used, when clinically indicated, to assess sensitization to specific protein components of soy (Gly m 4, Gly m 5, and Gly m 6) and peanut (Ara h 1, Ara h 2, Ara h 3, Ara h 8, and Ara h 9). Sensitization was defined as sIgE >0.10 kU_A_/L, corresponding to the analytical detection limit of the assay, to maximize sensitivity for detecting low-level IgE responses [38,39].

#### 2.3.3. Oral Food Challenges

OFCs were performed as part of routine clinical practice, and outcomes were analyzed retrospectively. Open OFCs were performed in a dedicated Day Hospital setting under the supervision of experienced pediatric allergists to confirm the diagnosis or reassess clinical reactivity when the last reaction had occurred >12 months earlier. Non-culprit OFCs were conducted when clinically indicated to differentiate clinically relevant co-allergy from asymptomatic co-sensitization. Challenges were conducted using cooked legumes, soy milk, or roasted peanuts according to a PRACTALL-based stepwise dose-escalation protocol, with incremental doses administered every 20–30 min up to an age-appropriate serving (maximum cumulative dose: 3 g of food protein) [28]. Patients were monitored throughout the procedure and for at least 2 h after the final dose. OFCs were discontinued upon the occurrence of objective or persistent IgE-mediated symptoms, including persistent oral allergy syndrome (OAS). Challenges were considered positive in such cases and negative when the full age-appropriate serving was tolerated without symptoms.

### 2.4. Oral Immunotherapy and Follow-Up Dietary Outcomes

OIT was offered as part of routine clinical practice following shared decision-making involving patients, caregivers, and the treating physician. Patients with contraindications (e.g., active EoE or uncontrolled asthma) were excluded. When multiple food allergies were present, treatment prioritization was guided by clinical relevance and family preference, with major allergens (e.g., milk, wheat, egg, or tree nuts) often addressed before legumes. Initiation and supervised dose escalation were conducted in a dedicated Day Hospital setting using cooked legumes, with a 2-h post-dose observation period. Dose increases were scheduled according to institutional practice and individual clinical course, typically over 6–12 months, starting from very small amounts of the culprit legume (e.g., approximately half to one pea, half to one chickpea, one lentil, half to one common bean, or 0.5 mL soy milk), corresponding to a few milligrams of legume protein, and gradually escalating to an age-appropriate serving size. Protein equivalents were estimated using standard food composition tables. Maintenance consisted of home administration of the highest tolerated dose achieved during supervised escalation, to be administered at least three times per week.

To minimize adverse events, doses were administered with food during periods of good health and optimal control of comorbidities. Patients were advised to avoid known cofactors, including strenuous physical activity within 1 h before and 4 h after dosing, as well as concomitant nonsteroidal anti-inflammatory drugs (NSAIDs) or proton pump inhibitors. Structured education was provided regarding recognition and management of adverse reactions and adherence to the prescribed regimen [29,30].

OIT was considered successful when patients tolerated a full age-appropriate serving of the legume without symptoms during regular ingestion, consistent with treatment-induced desensitization. Following successful OIT completion, regular ingestion was recommended to maintain desensitization. Patients still undergoing OIT who had not yet reached the full target dose at the last follow-up were on a lower partial maintenance dose.

Caregivers were contacted by telephone to obtain updated information on dietary intake and ongoing dietary restrictions. Current regular asymptomatic ingestion at follow-up was defined as ongoing ingestion of an age-appropriate serving without symptoms, documented in medical records and/or caregiver reports, either after OIT completion or following a period of avoidance. This real-world dietary outcome does not imply sustained unresponsiveness or permanent tolerance. In a subset of post-avoidance cases, asymptomatic ingestion was supported by a negative OFC followed by successful home reintroduction.

### 2.5. Definitions of Legume Co-Sensitization and Co-Allergy

- Co-sensitization was defined as IgE sensitization (SPT or PbP ≥ 3 mm and/or sIgE > 0.10 kU_A_/L) to one or more additional legumes in the absence of clinical symptoms upon exposure.
- Co-allergy was defined as a consistent clinical history of an immediate allergic reaction and/or a positive OFC to at least one additional legume, along with IgE sensitization to that same legume [26,40].
- Immunologic cross-reactivity was not directly assessed in this study.

### 2.6. Statistical Analysis

Statistical analyses were performed using SAS version 9.4 (SAS Institute, Cary, NC, USA). Continuous variables are expressed as medians with interquartile ranges (IQR), and categorical variables as counts and percentages. Group comparisons were performed using the chi-square test or Fisher’s exact test for categorical variables and the Kruskal–Wallis test for continuous variables. Statistical significance was set at *p* < 0.05.

## 3. Results

### 3.1. Demographic Characteristics and Comorbidities

Fifty-five patients met the inclusion criteria. The demographic and clinical characteristics of the study cohort are summarized in Table 1. The cohort was predominantly male, with a median age of 8 years (IQR 4–13). A family history of atopy was common, and all patients had at least one atopic comorbidity, with concomitant IgE-mediated food allergies being almost universal. Respiratory and cutaneous atopic diseases were frequent, and aeroallergen sensitization was detected in 80% of patients, predominantly to pollens, particularly grass pollen (Figure S1). EoE was identified in one patient who also had celiac disease. Four patients had a history of liver transplantation and subsequently developed TAFA under tacrolimus-based immunosuppression. Overall, non-atopic comorbidities were less frequent.

### 3.2. Clinical History and Culprit Legumes

Median age at first legume reaction was 18 months (IQR 9–48). Among children whose first legume reaction occurred before 5 years of age, 42% reacted within the first year of life and 73% by 24 months of age. Initial manifestations were predominantly mucocutaneous (38/55, 69.1%), particularly urticaria/angioedema (26/55, 47.3%) and OAS (11/55, 20.0%). Gastrointestinal symptoms (8/55, 14.5%) included nausea, vomiting, and abdominal pain. Anaphylaxis occurred in 7/55 (12.7%), whereas isolated respiratory symptoms were rare (2/55, 3.6%).

Approximately half of the initial reactions (28/55, 50.9%) were managed without pharmacological treatment. Among treated cases, antihistamines were the most frequently administered medication, whereas intramuscular epinephrine was used in one case. No age-related differences in clinical presentation were observed (Table S1).

Peas were the most common trigger at onset (17/55, 30.9%), followed by lentils (15/55, 27.3%), chickpeas (8/55, 15.5%), common beans (6/55, 10.9%), and soy (4/55, 7.3%). Peanut and faba bean were reported as initial legume triggers in one patient each (1.8%); in both cases, chickpea allergy was diagnosed within weeks at the same age. In three patients, the culprit legume remained unidentified due to mixed-legume preparations (soups, *n* = 2; prepackaged snacks, *n* = 1); these children were subsequently diagnosed with multiple legume allergies.

Among liver-transplant recipients who developed TAFA under tacrolimus-based immunosuppression (*n* = 4), legume allergy onset occurred a median of 22 months (IQR 8–39) post-transplantation, at a median age of 36 months (IQR 21–60). Lentils were the primary legume trigger (2/4), followed by common beans and peas (1/4 each). In one patient, legume allergy was the first FA. Clinical presentation at legume allergy onset mirrored that of the overall cohort, with predominantly mucocutaneous manifestations and one case of anaphylaxis (Table 2).

### 3.3. Skin Testing and Molecular Profiling

Skin test results and molecular profiles of the index legumes and co-allergies are summarized in Table 3. PbP yielded larger median wheal diameters than standardized SPT, particularly for pea (7.0 vs. 1.5 mm) and lentil (8.5 vs. 2.0 mm). Soy-allergic patients (*n* = 8) showed minimal skin reactivity despite detectable soy-specific IgE (median 1.7 kU_A_/L); CRD revealed predominant sensitization to Gly m 4 (median 26.6 kU_A_/L).

### 3.4. Non-Index Legumes Co-Allergies: Peanut, Faba Bean, and Lupine

Co-allergies to non-index legumes were identified in 17/55 patients (30.9%). Peanut was the most prevalent (14/55, 25.5%), followed by faba bean (3/55, 5.5%). The single patient with lupine co-allergy (1/55, 1.8%) also had concomitant peanut allergy.

Peanut allergy was diagnosed based on a consistent clinical history, including four cases of anaphylaxis, and was supported by IgE sensitization in 11/14 patients. In three cases, the diagnosis was confirmed by a positive OFC, including one case of anaphylaxis during the challenge. Molecular profiling revealed predominant Ara h 1 sensitization (median 1.4 kU_A_/L), whereas Ara h 2 levels were generally low (Table 3).

In contrast, co-allergies to faba bean (*n* = 3) and lupine (*n* = 1) were diagnosed based on clinical history and PbP testing (Table 3). Anaphylaxis occurred in one of three patients with faba bean allergy.

### 3.5. Concomitant IgE-Mediated Food Allergies

**Figure 2:**
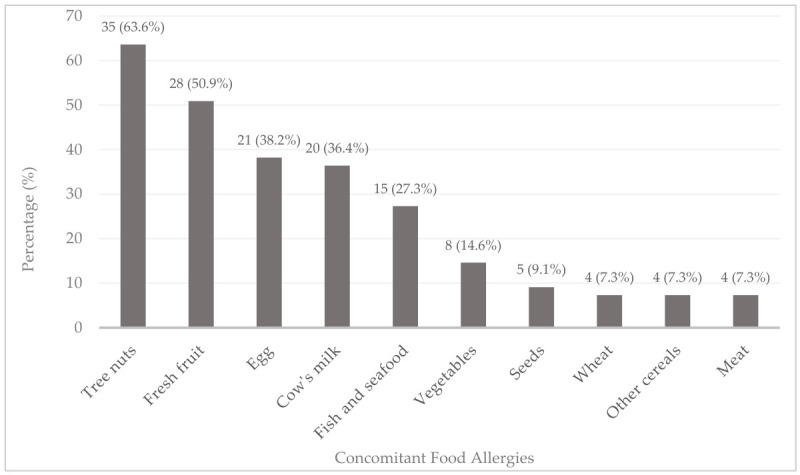
Concomitant food allergies in the study population. Values are presented as *n* (%).

Nearly all patients had at least one additional IgE-mediated FA. Tree nuts and fresh fruits were the most frequent concomitant allergens, followed by hen’s egg and cow’s milk, whereas other food groups were less commonly involved (Figure 2). Hazelnut predominated among tree nuts, and kiwifruit predominated among fresh fruits (Table S2).

The median age at onset of any FA was 9 months (IQR 6–24). Cow’s milk was the most common initial trigger (17/55, 30.9%), followed by legumes (15/55, 27.3%) and hen’s egg (10/55, 18.2%). Less frequent first eliciting foods included tree nuts (5/55, 9.1%), wheat (3/55, 5.5%), and fresh fruits (2/55, 3.6%). Single cases of fish (tuna) and meat (chicken) were reported as initial triggers, while in one patient the primary eliciting food could not be determined due to multiple reactions and limited parental recall.

### 3.6. Frequency of Legume Allergies and Patterns of Clinical Co-Allergy

**Figure 3:**
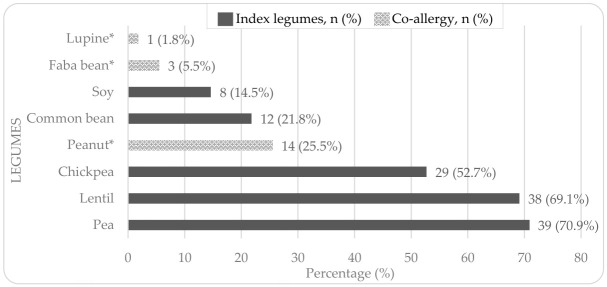
Frequency of legume allergies in the study cohort, including non-index legumes. Note: * Peanut, faba bean, and lupine were included only as co-allergies.

Pea was the predominant legume allergy in the cohort, followed by lentil and chickpea, whereas common bean and soy were less frequently observed (Figure 3).

Patterns of clinical co-allergy among legumes are summarized in Table 4. High co-allergy rates were observed among pea, lentil, and chickpea (56–86%), with the highest rate observed between chickpea and lentil (86%). Common bean showed high co-allergy rates with lentil (83%) and moderate rates with pea and chickpea (67% each). Soy exhibited lower overall co-allergy rates, most frequently with lentil and chickpea (50% each).

Among non-index legumes, peanut showed the highest co-allergy rate with pea (79%), followed by lentil and chickpea (57% each). Faba bean co-allergy was primarily observed with pea (67%), whereas lupine (*n* = 1) showed co-allergy only with peanut and soy (Table 4).

### 3.7. Oral Food Challenges and Oral Immunotherapy Outcomes

Thirty-four OFCs to index legumes were performed to confirm the diagnosis (*n* = 4, all positive), differentiate between co-sensitization and co-allergy (*n* = 17, 2 positive), or reassess clinical reactivity to the initial culprit legume (*n* = 13, 2 positive). Overall, 26/34 (76.5%) OFCs were negative and 8/34 (23.5%) were positive. Not all negative OFCs were followed by home reintroduction. One patient reported OAS during home reintroduction of pea despite prior tolerance of the full target dose during a supervised OFC. Positive challenges most frequently involved chickpea (4/8), and persistent OAS was the predominant presentation (5/8). Anaphylaxis occurred during a single pea OFC. The remaining positive challenges involved lentil (*n* = 1, persistent OAS), common bean (*n* = 1, nausea and vomiting), and soy (*n* = 1, persistent OAS).

Among non-index legumes, 17 peanut OFCs were performed; 3/17 (17.6%) were positive, including one case of anaphylaxis, whereas 14/17 ruled out clinically relevant peanut allergy. Three additional OFCs to other non-index legumes (green bean, *n* = 2; lupine, *n* = 1) were performed to exclude clinically relevant allergy, and all were negative. Detailed OFC outcomes by legume are shown in Table S3.

Sixteen OIT protocols were initiated in 15 patients, including one patient who completed chickpea OIT and subsequently started a pea OIT protocol that remained ongoing at last follow-up. Overall, 5/16 protocols (31.3%) reached the full target maintenance dose after a median duration of 24 months (median age 9 years, IQR 5–9). Six of 16 protocols (37.5%) remained ongoing at a lower, partial maintenance dose after a median duration of 30 months (median age 9.5 years, IQR 6–14), with tolerated doses of 2 chickpeas (*n* = 1; corresponding to ~60 mg of chickpea protein), a median of 6.5 peas (*n* = 4; ~100 mg of pea protein), and 2 mL soy milk (*n* = 1; ~60 mg of soy protein). The remaining 5/16 protocols (31.3%) were discontinued due to persistent OAS.

**Figure 4:**
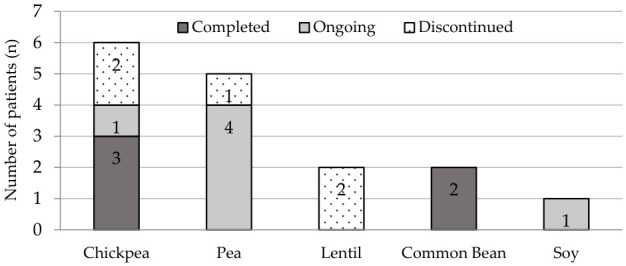
Outcomes of OIT for index legume allergy in the study cohort. Note: Bars represent the number of patients per legume who completed, were undergoing, or discontinued OIT.

Outcomes differed by legume species: all common bean OITs were completed (2/2), both lentil OITs were discontinued (2/2), chickpea OITs showed variable outcomes, pea OITs were mostly ongoing (4/5), and the single soy OIT remained ongoing (Figure 4).

Among non-index legumes, four patients underwent peanut OIT: 2/4 reached the full target maintenance dose after 24 months (ages 5 and 6 years), whereas 2/4 discontinued due to adverse reactions (OAS in both cases, with abdominal pain in one). No OIT was initiated for faba bean or lupine.

### 3.8. Species-Specific Clinical Characteristics and Subgroup Analysis

Clinical characteristics and follow-up dietary outcomes by legume species are shown in Table 5. Soy exhibited the highest proportion of single-legume allergy (3/8, 37.5%), whereas multi-legume allergy predominated among the other index legumes. Concomitant food allergies were nearly universal (96.6–100%). Pollen sensitization was common across index legumes (62.5–83.3%) and was observed in all patients with faba bean or lupine co-allergy, although numbers were small. Ingestion was the predominant route of exposure.

Among index legumes, anaphylaxis occurred most frequently in pea allergy (8/39, 20.5%), accounting for half of all index-legume anaphylaxis cases (8/16). Among co-allergies, anaphylaxis was observed only in peanut (5/14, 35.7%) and faba bean (1/3, 33.3%).

Overall, 18/55 patients (32.7%) reported current regular asymptomatic ingestion of at least one index legume at follow-up, including 5 after OIT and 13 following a period of avoidance. At the legume level, asymptomatic ingestion involved 24 index legumes: 5 after OIT (chickpea *n* = 3; common bean *n* = 2) and 19 after avoidance. Among post-avoidance cases, 6 were supported by a negative OFC followed by successful home reintroduction, and the median interval between the last reaction and reintroduction was 5 years (IQR 3–9). Rates varied across legume species. Common bean showed the highest proportion of current asymptomatic ingestion (6/12, 50.0%), including both OIT-induced desensitization (*n* = 2) and reintroduction after avoidance (*n* = 4). In contrast, lentil showed the lowest rate (4/38, 10.5%), with all cases occurring after avoidance. For chickpea and common bean, asymptomatic ingestion occurred both after OIT (3/5 and 2/6, respectively) and after avoidance (2/5 and 4/6, respectively), whereas for pea, lentil, and soy it occurred exclusively following avoidance (Table S4).

At the patient level, 12/55 (21.8%) reported asymptomatic ingestion of the initial culprit legume and 6/55 (10.9%) reported asymptomatic ingestion of legumes implicated in subsequent reactions, whereas 37/55 (67.3%) continued strict avoidance. As multiple legume allergies were common (*n* = 41), species-level counts exceeded the number of individual patients. No statistically significant association was observed between age at onset and current asymptomatic ingestion (all *p* > 0.05).

Among non-index legumes, current asymptomatic ingestion of peanut was reported in three patients, following OIT completion (*n* = 2) or after 10 years of avoidance (*n* = 1), whereas patients with faba bean or lupine co-allergies remained on strict avoidance.

The characteristics of patients with single versus multiple legume allergies are summarized in Table 6. Patients with single-legume allergy (14/55, 25.5%) had a later age at onset than those with multiple legume allergies (median 54 vs. 15 months; *p* = 0.013) and were more likely to have a history of liver transplantation (21% vs. 2%; *p* = 0.047). A non-significant trend toward male predominance was observed among patients with single-legume allergy (*p* = 0.059).

Severity of the initial reaction (anaphylaxis vs. mild-to-moderate) did not differ between groups. No significant differences were observed in demographic or laboratory parameters, aeroallergen sensitization, or rates of concomitant FA. A non-significant trend toward a higher prevalence of cow’s milk allergy was noted in the single-legume group (57.1% vs. 29.3%; *p* = 0.062; Table S5).

## 4. Discussion

This retrospective single-center study provides a comprehensive characterization of pediatric IgE-mediated legume allergy in an Italian tertiary allergy setting by integrating clinical history, sensitization profiles, CRD data when available, OFC outcomes, and real-world management data, including OIT. Together, these complementary dimensions allowed us to identify culprit legumes, distinguish clinically relevant allergy from sensitization alone, reassess clinical reactivity over time, and describe management strategies beyond avoidance.

Overall, legume allergy in this cohort showed early onset, male predominance, a high burden of atopy, frequent multi-legume involvement, and a high prevalence of concomitant FA, consistent with previous Mediterranean pediatric cohorts [17,18]. Pea was the most frequently implicated index legume, followed closely by lentil, and was associated with the highest proportion of index-legume anaphylaxis. Species-specific differences also emerged in sensitization patterns, OFC outcomes, OIT feasibility, and follow-up dietary outcomes.

Although legumes followed a prior FA in most cases—particularly cow’s milk—they represented the index allergen in approximately one-quarter of patients, supporting their role as potential primary sensitizers in early childhood. Together with the high prevalence of multi-legume allergy (74.5%) and concomitant FA (96.4%), this finding underscores the clinical and nutritional complexity of pediatric legume allergy, particularly in children with multiple dietary restrictions, in whom legume allergy may further limit nutritional diversity and reduce available plant-based protein alternatives.

Patients with single-legume allergy (25.5%) exhibited a significantly later onset (median 54 vs. 15 months) and were more likely to have a history of liver transplantation (21% vs. 2%). Although statistically significant, the latter association was based on very small absolute numbers and should therefore be interpreted as exploratory. Children with TAFA showed a later onset of legume allergy (median 36 months) under tacrolimus-based immunosuppression and predominantly single-legume involvement, consistent with previous reports suggesting that tacrolimus-associated immune dysregulation may contribute to *de novo* sensitization to commonly consumed foods [33,34,35]. Taken together, these observations suggest that TAFA may represent a distinct but uncommon phenotype within pediatric legume allergy, although further evaluation in larger cohorts of liver transplant recipients is needed.

In contrast to reports from other Mediterranean countries, where lentil is generally reported as the predominant legume allergen [17,18,25], pea was the most frequently implicated legume in our cohort, although only slightly more prevalent than lentil, likely reflecting regional dietary patterns and evolving exposure to pea-derived ingredients. Importantly, previous Mediterranean cohorts also identified pea allergy as a clinically relevant and widespread component of pediatric legume allergy, even when less frequent than lentil or chickpea allergy [17,18,25]. In our cohort, the relevance of pea allergy extended beyond its frequency, as it was associated with the highest proportion of index-legume anaphylaxis cases. Although this observation should be interpreted in light of the small number of severe reactions, it supports careful consideration of pea in diagnostic evaluation and individualized risk assessment.

One episode of anaphylaxis occurred after ingestion of undeclared pea protein in prepackaged ice cream in a child with multiple legume allergies, underscoring the risks posed by hidden legume ingredients in processed foods and the potential for severe reactions following unintended exposure. Beyond this case, clinical history identified several real-world exposure scenarios, including mixed-legume soups, hummus, soy-based beverages, legume-based snack products, and processed foods containing legume-derived ingredients, such as lentil flour and pea protein. Although descriptive, these observations may help guide diagnostic history-taking by emphasizing the need to specifically assess hidden legume-derived ingredients, particularly as legume protein isolates are increasingly used in plant-based and processed foods. In settings where labeling regulations do not require declaration of non-priority legumes, these exposures may remain difficult to identify, limiting opportunities for effective avoidance and risk reduction. These findings support heightened clinical awareness of certain non-priority legumes, including pea, in Mediterranean settings and suggest potential gaps in current labeling frameworks in capturing region-specific allergen risks [4,9,11,12,13,14].

From a diagnostic perspective, cooked or canned legumes and soy milk were used according to the patient’s clinical history and suspected culprit food. In this context, the discrepancy between commercial extract-based SPT and PbP testing, particularly for pea, may reflect underrepresentation of relevant allergens in commercial extracts and should be interpreted cautiously, while suggesting the need for further evaluation of species-specific diagnostic approaches in legume allergy. Soy allergy exhibited a distinct pattern, with the highest proportion of single-legume allergy among index legumes and predominant Gly m 4 sensitization, consistent with a PR-10-mediated, pollen-related phenotype in a highly pollen-sensitized cohort. In contrast to most legumes, soy showed minimal skin reactivity even with PbP, consistent with the labile nature of PR-10 proteins. Overall, these findings reinforce the complementary diagnostic value of PbP and CRD, when available, while underscoring species-specific patterns of sensitization.

The high degree of co-allergy observed among pea, lentil, and chickpea may reflect underlying cross-reactivity driven by shared seed storage proteins within the *Fabaceae* family; however, this mechanism was not directly assessed in our cohort [15,23]. Co-allergy was defined conservatively, requiring either a consistent clinical history or a positive OFC together with IgE sensitization, thereby excluding asymptomatic co-sensitization. Although not all cases were challenge-confirmed, this approach reflects real-world clinical practice.

Reliance on sensitization testing alone may overestimate clinically relevant allergies, potentially leading to unnecessary avoidance of botanically related legumes, with consequent dietary restrictions and negative impacts on quality of life. The high proportion of negative OFCs supports the role of challenge-based reassessment in refining diagnosis and guiding individualized dietary management in multi-sensitized patients. However, negative OFC outcomes did not consistently lead to home reintroduction, often reflecting patient or caregiver preferences rather than clinical reactivity, highlighting a gap between OFC outcomes and real-world dietary practices.

Among non-index legumes, peanut was the most frequent co-allergen (25.5%). Molecular profiling showed predominant Ara h 1 sensitization with low levels of Ara h 2 sIgE. Given that Ara h 2 is commonly associated with primary, severe peanut allergy, this pattern may reflect cross-sensitization to homologous 7S globulins (vicilins), including Ara h 1, rather than primary peanut allergy in some children [23]. The high clinical co-allergy observed between pea and peanut (79%) is consistent with shared protein homology, although mechanistic conclusions cannot be definitively established from these data. These findings support the clinical utility of CRD in evaluating peanut co-allergy in patients with multiple legume allergies.

Less common legumes, such as faba bean and lupine, were evaluated as non-index co-allergies by study design and were typically observed within broader multiple-legume allergy phenotypes, possibly reflecting a higher overall atopic burden [18]. Despite the low prevalence of faba bean co-allergy (5.5%), one case of anaphylaxis was documented in our cohort, indicating that clinically relevant reactions may occur even with less commonly consumed legumes.

In this real-world cohort, OIT prioritization often favored major allergens (milk, wheat, egg, and tree nuts), influencing both the timing and selection of legume OIT. Desensitization through OIT appeared feasible, although outcomes varied across legume species. OAS was a frequent limiting factor, particularly for lentil, whereas OIT completion rates were higher for common bean. However, species-specific OIT outcomes should be interpreted cautiously given the very small number of treated patients for each legume species; therefore, the observed differences should be considered exploratory and hypothesis-generating. Overall, these findings highlight the heterogeneity of legume allergies and support the need for legume-specific OIT protocols [30,32].

Follow-up dietary outcomes varied across legume species and reflected two distinct clinical scenarios: post-OIT desensitization and asymptomatic reintroduction following avoidance, the latter being compatible with a possible spontaneous resolution. Asymptomatic reintroduction following avoidance was more frequent for common bean than for lentil, consistent with previous reports suggesting a more favorable natural history for common bean [10]. Overall, 32.7% of patients reported current regular asymptomatic ingestion of at least one index legume at follow-up, including 21.8% who resumed ingestion of the initial culprit legume. However, these findings reflect real-world dietary exposure rather than systematically challenge-confirmed tolerance, as only a minority of cases (6/24) were supported by a negative OFC followed by successful home reintroduction. Outcomes following OIT reflect treatment-induced desensitization dependent on continued allergen exposure, whereas outcomes after avoidance may reflect spontaneous resolution but were not systematically confirmed by OFC. Given the limited number of patients completing OIT, its contribution to long-term tolerance development cannot be determined from our data.

Taken together, these findings suggest that legume allergy may persist in a substantial proportion of children, with prognosis varying by species and potential implications for long-term dietary management. Larger prospective studies are needed to clarify the contribution of OIT to tolerance development in pediatric legume allergy.

Our study is limited by its retrospective, single-center design in a tertiary referral setting, which may introduce referral bias and limit generalizability. In particular, the very high prevalence of atopic comorbidities and concomitant FA likely reflects the referral characteristics of the cohort and may not capture the full spectrum of pediatric legume allergy observed in community-based populations, however, as the tertiary-center setting allows the referral and evaluation of the majority of pediatric patients with suspected or confirmed legume allergy in our area the cohort may still provide a representative picture of an expanded real-world population of children with clinically relevant legume allergy. The relatively small sample size for certain legume species and subgroups reduces statistical power; accordingly, species-specific and subgroup findings should be considered exploratory and hypothesis-generating. In addition, testing for co-allergy was guided by clinical indication rather than performed through a systematic screening protocol for all legumes in all patients, which may have influenced the observed co-allergy patterns. OFCs were conducted using an open rather than double-blind design, consistent with real-world practice but potentially susceptible to expectation bias. Although a low analytical threshold for sIgE (>0.10 kU_A_/L) was used to increase sensitivity, laboratory results were interpreted in a clinical context. Both diagnosis and definition of clinical co-allergy required a compatible clinical history and/or a positive OFC, minimizing misclassification and reducing the risk of overestimating clinically relevant allergies. Functional assays, such as basophil activation tests or inhibition assays, were not performed; therefore, potential cross-reactivity mechanisms among legumes can only be inferred from clinical co-allergy patterns and sensitization profiles. Follow-up dietary outcomes should be interpreted with caution, as most were based on caregiver-reported ingestion and were not systematically confirmed by OFC, potentially overestimating true clinical tolerance. These outcomes may also reflect caregiver or patient preferences and adherence to dietary recommendations. Sustained unresponsiveness cannot be inferred, as no post-OIT avoidance phase with confirmatory OFC was performed.

Despite these limitations, this study represents, to our knowledge, the first comprehensive Italian characterization of pediatric legume allergy, integrating clinical history, OFC outcomes, CRD, and real-world OIT data within a Mediterranean pediatric allergy setting. Prospective multicenter studies are warranted to better define the natural history of pediatric legume allergy, refine risk stratification, optimize legume-specific desensitization protocols, and inform evidence-based dietary counseling and allergen prioritization strategies.

## 5. Conclusions

Pediatric legume allergy in Italy is characterized by early onset, a robust atopic background, and frequent multi-legume involvement, posing significant challenges for diagnosis and dietary management. Single-legume allergy was less common, with some cases occurring in the context of TAFA.

In this cohort, pea allergy was associated with the highest proportion of severe reactions, including anaphylaxis, underscoring the clinical relevance of selected non-priority legumes in Mediterranean populations. In the context of the increasing use of legume-derived ingredients in processed foods, current labeling frameworks may not fully capture region-specific risks associated with these legumes.

These findings further support improved risk stratification, individualized management, and a risk-based approach to allergen prioritization. Larger prospective, multicenter studies are warranted to better define the natural history of pediatric legume allergy, elucidate the mechanisms underlying cross-reactivity, optimize legume-specific desensitization protocols, and inform evidence-based updates to allergen labeling policies.

## Figures and Tables

**Table 1:** Demographics and comorbidities of the study population.

	Study Population (*N* = 55)
Male gender, *n* (%)	35 (63.6)
Age at inclusion, years, median (IQR)	8 (4–13)
Family history of atopy, *n* (%)	44 (80.0)
Atopic comorbidities, *n* (%)	55 (100)
Concomitant food allergies	53 (96.4)
Allergic rhinitis	39 (70.9)
AD	37 (67.3)
Asthma	28 (50.9)
EoE	1 (1.8)
Drug hypersensitivity	6 (10.9)
Other comorbidities, *n* (%)	17 (30.9)
Celiac disease	4 (7.3)
History of liver transplantation	4 (7.3)
Gastroesophageal reflux	3 (5.4)
Autism spectrum disorder	3 (5.4)
CSU	2 (3.6)

Note: IQR, interquartile range; AD, atopic dermatitis; EoE, eosinophilic esophagitis; CSU, chronic spontaneous urticaria.

**Table 2:** Clinical characteristics of food and legume allergy in pediatric liver transplant recipients (N = 4).

Patient	First FA (Clinical Presentation)	Age at FA Onset (Interval From Tx) °	First LA (Clinical Presentation)	Age at LA Onset (Interval From Tx) °
1	Lentil (anaphylaxis)	24 (9)	Lentil (anaphylaxis)	24 (9)
2	Cow’s milk (anaphylaxis)	25 (13)	Lentil (OAS)	48 (34)
3	Chicken (U/A)	60 (31)	Common bean (U/A)	72 (43)
4	Egg (anaphylaxis)	11 (1)	Pea (angioedema)	17 (7)

Note: ° Ages and intervals are expressed in months; time from transplantation is shown in parentheses. Tx, transplantation; FA, food allergy; LA, legume allergy; OAS, oral allergy syndrome; U/A, urticaria/angioedema.

**Table 3:** Skin test results and legume-specific IgE levels, including CRD as applicable.

Legume (*n*)	SPT °, mm	PbP °, mm	sIgE °, kUA/L	CRD °, kU_A_/L
Pea (39)	1.5 (0–3.0)	7.0 (4.0–10.0)	3.8 (1.1–9.1)	–
Lentil (38)	2.0 (2.0–3.0)	8.5 (5.0–10.0)	4.2 (1.6–12.8)	–
Chickpea (29)	2.0 (2.0–3.0)	5.0 (4.0–6.5)	5.4 (2.0–16.5)	–
Common bean (12)	NA	2.0 (0.5–2.8)	0.9 (0.3–9.8)	–
Soy (8)	0 (0–1.0)	0 (0–3.0)	1.7 (0.8–6.1)	*Gly m 4*: 26.6 (0.0–76.6); *Gly m 5*: 0.2 (0.1–0.3); *Gly m 6*: 0.6 (0.3–1.5)
**Co-allergies**				
Peanut (14)	NA	4.0 (2.0–6.0)	5.5 (0.8–32.0)	*Ara h 1*: 1.4 (0.0–16.0); *Ara h 2*: 0.2 (0.0–0.6); *Ara h 3*: 0.2 (0.0–1.4); *Ara h 8*: 0.1 (0.0–2.3); *Ara h 9*: 0.0 (0.0–3.2)
Faba bean (3)	NA	5.0 (4.0–9.0)	NA	–
Lupine (1)	NA	4.0	NA	–

Note: ° Continuous variables are reported as median (IQR). SPT, skin prick test; PbP, prick-by-prick; sIgE, serum-specific IgE; CRD, component-resolved diagnosis; NA, not available. A dash (–) indicates not applicable.

**Table 4:** Clinical co-allergy patterns among legumes in the study cohort.

	Pea	Lentil	Chickpea	Peanut	Common Bean	Soy	Faba Bean	Lupine
Pea (n = 39)		29 (74%)	22 (56%)	11 (28%)	8 (21%)	3 (8%)	2 (5%)	0
Lentil (n = 38)	29 (76%)		25 (66%)	8 (21%)	10 (26%)	4 (11%)	1 (3%)	0
Chickpea (n = 29)	22 (76%)	25 (86%)		8 (28%)	8 (28%)	4 (14%)	1 (3%)	0
* Peanut (n = 14)	11 (79%)	8 (57%)	8 (57%)		3 (21%)	3 (21%)	0	1 (7%)
Common bean (n = 12)	8 (67%)	10 (83%)	8 (67%)	3 (25%)		1 (8%)	0	0
Soy (n = 8)	3 (38%)	4 (50%)	4 (50%)	3 (38%)	1 (13%)		0	1 (13%)
* Faba bean (n = 3)	2 (67%)	1 (33%)	1 (33%)	0	0	0		0
* Lupine (n = 1)	0	0	0	1 (100%)	0	1 (100%)	0	

Note: Rows indicate patients allergic to the respective legume; columns indicate co-allergy to other legumes. Values are expressed as *n* (%) of patients with co-allergy. Percentages were calculated using the number of patients allergic to the legume indicated in each row as the denominator. * Peanut, faba bean, and lupine were observed only as co-allergies by study design. 0% 1–40% 41–65% 66–99% 100%.

**Table 5:** Clinical characteristics and follow-up dietary outcomes by legume species.

	Index Legumes					Non-Index Legumes		
	Pea (N = 39)	Lentil (N = 38)	Chickpea (N = 29)	Common Bean (N = 12)	Soy (N = 8)	Peanut (N = 14)	Faba Bean (N = 3)	Lupine (N = 1)
Single LA, *n*/N (%)	6/39 (15.4)	3/38 (7.9)	1/29 (3.5)	1/12 (8.3)	3/8 (37.5)	–	–	–
Concomitant FA, *n* (%)	38 (97.4)	37 (97.4)	28 (96.6)	12 (100)	8 (100)	14 (100)	3 (100)	1 (100)
Pollen sensitization, *n* (%)	26 (66.7)	28 (73.7)	22 (75.9)	10 (83.3)	5 (62.5)	10 (71.4)	3 (100)	1 (100)
Route of exposure, *n* (%)								
Ingestion	38 (97.4)	33 (86.8)	28 (96.6)	12 (100)	8 (100)	12 (85.7)	2 (66.7)	1 (100)
Contact	0	3 (7.9)	0	0	0	2 (14.3)	1 (33.3)	0
Inhalation	0	2 (5.3)	0	0	0	0	0	0
Anaphylaxis (ever), *n*/N (%)	8/39 (20.5)	4/38 (10.5)	3/29 (10.3)	1/12 (8.3)	0	5/14 (35.7)	1/3 (33.3)	0
Current asymptomatic ingestion *, *n*/N (%)	7/39 (17.9)	4/38 (10.5)	5/29 (17.2)	6/12 (50.0)	2/8 (25.0)	3/14 (21.4)	0	0

Note: Values are expressed as *n* (%) unless otherwise specified. Percentages were calculated using the number of patients allergic to each legume (N in column headers) as the denominator. LA, legume allergy; FA, food allergy. A dash (–) indicates not applicable. * Current regular asymptomatic ingestion was defined as ongoing ingestion of an age-appropriate serving without symptoms at follow-up, either after OIT completion or after a period of avoidance. For chickpea and common bean, this included both post-OIT desensitization and post-avoidance asymptomatic reintroduction.

**Table 6:** Characteristics of patients with single versus multiple legume allergies.

	Study Cohort (N = 55)	Single LA (N = 14)	Multiple LA (N = 41)	*p*-Value
Male gender, *n* (%)	35 (63.6)	12 (85.7)	23 (56.1)	0.0587
Age at inclusion, y, median (IQR)	8 (4–13)	8 (7–11)	7 (4–14)	0.9306
Age at FA onset, mo, median (IQR)	9 (6–24)	12.5 (8–27)	8 (6–18)	0.1325
Age at LA onset, mo, median (IQR)	18 (9–48)	54 (17–72)	15 (8–36)	**0.0131**
Concomitant FA, *n* (%)	53 (96.4)	13 (92.9)	40 (97.6)	*0.4478*
Pollen sensitization, *n* (%)	40 (72.7)	9 (64.3)	31 (75.6)	*0.4926*
Family history of atopy, *n* (%)	44 (80.0)	11 (78.6)	33 (80.5)	1.0000
Total IgE, kU/L, median (IQR)	552 (273–1660)	1353 (267–2615)	514 (282–1204)	0.2690
Clinical presentation at onset, *n* (%)				0.8651 *
Anaphylaxis	7 (12.7)	2 (14.3)	5 (12.2)	
GI	8 (14.6)	2 (14.3)	6 (14.6)	
Mucocutaneous	38 (69.1)	10 (71.4)	28 (68.3)	
Respiratory	2 (3.6)	0	2 (4.9)	
Atopic comorbidities, *n* (%)				
Allergic rhinitis	39 (70.9)	9 (64.3)	30 (73.2)	0.5186
AD	37 (67.3)	9 (64.3)	28 (68.3)	1.0000
Asthma	28 (50.9)	7 (50.0)	21 (51.2)	0.9372
EoE	1 (1.8)	0	1 (2.4)	1.0000
Other comorbidities, *n* (%)				
Drug hypersensitivity	6 (10.9)	1 (7.1)	5 (12.2)	1.0000
Celiac disease	4 (7.3)	0	4 (9.8)	0.5624
Liver transplantation	4 (7.3)	3 (21.4)	1 (2.4)	**0.0467**

Note: y, years; mo, months; IQR, interquartile range; FA, food allergy; LA, legume allergy; GI, gastrointestinal; AD, atopic dermatitis; EoE, eosinophilic esophagitis. Bold values indicate statistical significance (*p* < 0.05). Continuous variables were compared using the Kruskal–Wallis test; categorical variables were compared using the chi-square test or Fisher’s exact test, as appropriate. * Comparison across clinical presentation categories (anaphylaxis, respiratory, GI, and mucocutaneous).

## Data Availability

The raw data supporting the conclusions of this article will be made available by the authors on request.

## Supplementary Materials

The following supporting information can be downloaded at https://www.mdpi.com/article/10.3390/nu18111810/s1, Figure S1. Aeroallergen sensitization profile in the study cohort; Table S1. Clinical manifestations at legume allergy onset according to age group; Table S2. Distribution of concomitant tree nut and fresh fruit allergies; Table S3. OFC outcomes by legume; Table S4. Follow-up dietary outcomes by index legume species; Table S5. Aeroallergen sensitization and concomitant food allergies in patients with single vs. multiple legume allergies.

## Abbreviations

The following abbreviations are used in this manuscript:

FA	Food allergy
LA	Legume allergy
OAS	Oral allergy syndrome
AD	Atopic dermatitis
EoE	Eosinophilic esophagitis
CSU	Chronic spontaneous urticaria
OFC	Oral food challenge
OIT	Oral immunotherapy
SPT	Skin prick test
PbP	Prick-by-prick
sIgE	Serum-specific IgE
TAFA	Transplant-acquired food allergy
