# Supplementary material for: IgE-Mediated Legume Allergy in Children: Insights from a Single-Center Experience in Italy
Source: Nutrients. 2026 Jun 4;18(11):1810. doi: 10.3390/nu18111810 (PMC13258717; doi:10.3390/nu18111810)
Supplement: Supplementary file 1 [file nutrients-18-01810-s001.zip › nutrients-4291457-supplementary.pdf]

nutrients MDPI

SUPPLEMENTARY MATERIALS

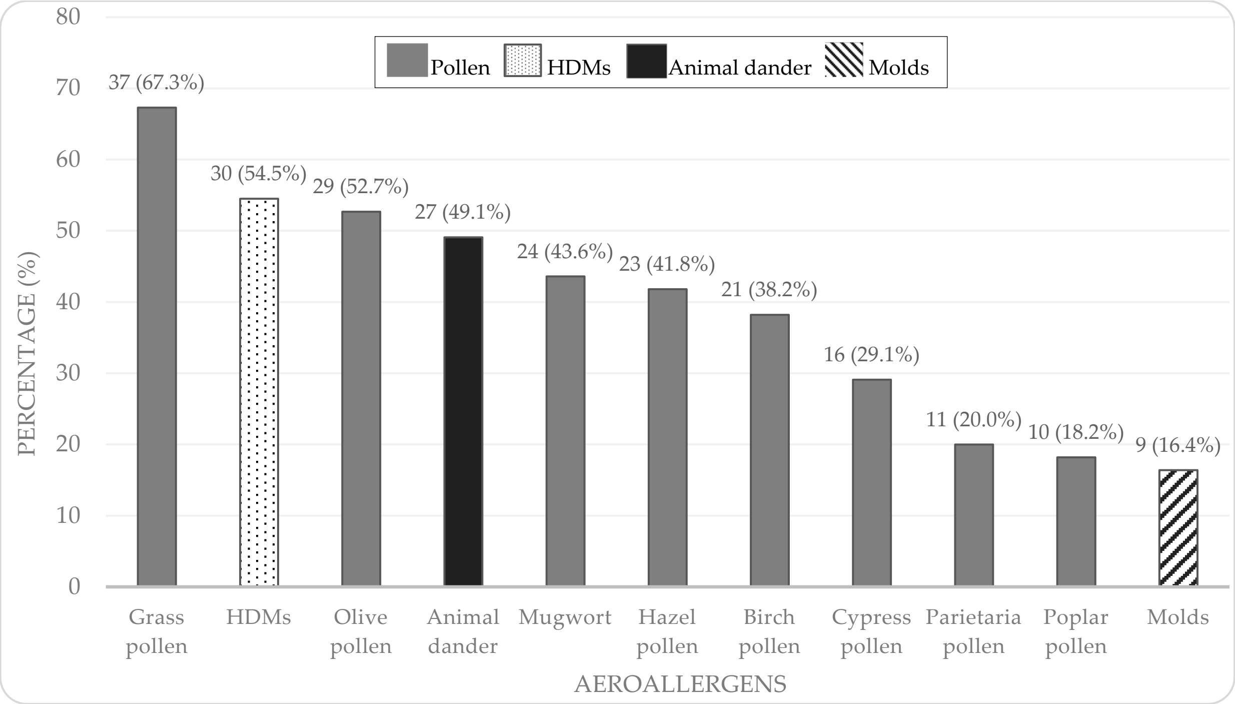

Figure S1. Aeroallergen sensitization profile in the study cohort (N = 55).

Prevalence of sensitization to major inhalant allergens assessed by skin prick test (SPT) and/or serum-specific IgE (sIgE). Data are presented as n (%). HDMs, house dust mites (*Dermatophagoides farinae, Dermatophagoides pteronyssinus*); animal dander includes cat, dog, and horse; molds include *Alternaria* and *Cladosporium*.

Table S1. Clinical manifestations at legume allergy onset according to age group.

Manifestation at LA onset	0–5 years (N = 48, 87.3%)	6–12 years (N = 4, 7.3%)	>12 years (N = 3, 5.5%)	Total (N = 55)	*p*-value
Mucocutaneous	33 (68.8)	3 (75.0)	2 (66.7)	38 (69.1)	0.3763
Gastrointestinal	7 (14.6)	1 (25.0)	–	8 (14.6)	
Anaphylaxis	7 (14.6)	–	–	7 (12.7)	
Respiratory	1 (2.1)	–	1 (33.3)	2 (3.6)	

Note: Data are presented as n (%). LA, legume allergy. A dash (–) indicates no cases. The p-value refers to the overall comparison across clinical manifestation categories by age group using Fisher's exact test.

Table S2. Distribution of concomitant tree nut and fresh fruit allergies.

Tree nuts (N = 35)	n (%)	Fresh fruits (N = 28)	n (%)
Hazelnut	16 (45.7)	Kiwifruit	12 (42.9)
Walnut	12 (34.3)	Peach	9 (32.1)
Cashew	8 (22.8)	Banana	4 (14.3)
Pistachio	7 (20.0)	Apple	3 (10.7)
Almond	7 (20.0)	Melon	2 (7.1)
Pine nut	3 (8.6)	Plum	2 (7.1)
		Avocado	1 (3.6)
		Pineapple	1 (3.6)

Note: Percentages were calculated within each food group.

Table S3. OFC outcomes by legume.

Legume	OFCs (n)	Positive, n (%)	Anaphylaxis (n)
**Index legumes (overall)**	**34**	**8 (23.5)**	**1**
Pea	7	1 (14.3)	1
Lentil	5	1 (20.0)	0
Chickpea	11	4 (36.4)	0
Common bean	7	1 (14.3)	0
Soy	4	1 (25.0)	0
**Non-index legumes (overall)**	**20**	**3 (15.0)**	**1**
Peanut	17	3 (17.6)	1
Green bean†	2	0	0
Lupine†	1	0	0

Note: Percentages were calculated per legume (row-wise). OFC, oral food challenge.

† Tested to distinguish co-sensitization from clinically relevant allergy.

Table S4. Follow-up dietary outcomes by index legume species.

Legume (N)	Current regular asymptomatic ingestion*, n/N (%)	After OIT (n)	After avoidance (n)	OFC-supported reintroduction° (n)
Pea (39)	7/39 (17.9)	0	7	2
Lentil (38)	4/38 (10.5)	0	4	1
Chickpea (29)	5/29 (17.2)	3	2	0
Common bean (12)	6/12 (50.0)	2	4	1
Soy (8)	2/8 (25.0)	0	2	2
**Total (index legumes)**	**24/126 (19.0)**	**5**	**19**	**6**

Note: * Current regular asymptomatic ingestion was defined as ongoing ingestion of an age-appropriate serving without symptoms at follow-up, based on medical records and/or caregiver reports. ° Among post-avoidance cases, 6/19 were supported by a negative OFC followed by successful home reintroduction. OIT, oral immunotherapy; OFC, oral food challenge.

Table S5. Aeroallergen sensitization and concomitant food allergies in patients with single versus multiple legume allergies.

	Study population (N = 55)	Single LA (N = 14)	Multiple LA (N = 41)	*p-value*
**Aeroallergen sensitization, n (%)**	44 (80.0)	10 (71.4)	34 (82.9)	*0.4429*
Pollen	40 (72.7)	9 (64.3)	31 (75.6)	*0.4926*
HDMs	30 (54.6)	9 (64.3)	21 (51.2)	*0.5371*
Animal dander	27 (49.1)	9 (64.3)	18 (43.9)	*0.2270*
Molds	9 (16.4)	3 (21.4)	6 (14.6)	*0.6782*
**Concomitant FA, n (%)**	53 (96.4)	13 (92.9)	40 (97.6)	*0.4478*
Tree nuts	35 (63.6)	8 (57.1)	27 (65.9)	*0.5586*
Fresh fruits	28 (50.9)	9 (64.3)	19 (46.3)	*0.3550*
Egg	21 (38.2)	5 (35.7)	16 (39.0)	*1.0000*
Cow's milk	20 (36.4)	8 (57.1)	12 (29.3)	*0.0612*
Fish and seafood	15 (27.3)	3 (21.4)	12 (29.3)	*0.7342*
Peanut	14 (25.5)	4 (28.6)	10 (24.4)	*0.7358*
Vegetables	8 (14.6)	3 (21.4)	5 (12.2)	*0.4054*
Seeds	5 (9.1)	2 (14.3)	3 (7.3)	*0.5924*
Wheat	4 (7.3)	–	4 (9.8)	*0.5624*
Other cereals	4 (7.3)	2 (14.3)	2 (4.9)	*0.2655*
Meat	4 (7.3)	2 (14.3)	2 (4.9)	*0.2655*

Note: Data are presented as n (%). HDMs, house dust mites; FA, food allergy; LA, legume allergy. A dash (–) indicates no cases. Categorical variables were compared using the chi-square test or Fisher's exact test, as appropriate.
